# Determinants of the accuracy of rapid diagnostic tests in malaria case management: evidence from low and moderate transmission settings in the East African highlands

**DOI:** 10.1186/1475-2875-7-202

**Published:** 2008-10-03

**Authors:** Tarekegn A Abeku, Mojca Kristan, Caroline Jones, James Beard, Dirk H Mueller, Michael Okia, Beth Rapuoda, Brian Greenwood, Jonathan Cox

**Affiliations:** 1 London School of Hygiene & Tropical Medicine, Keppel Street, London, WC1E 7HT, UK; 2 National Malaria Control Programme, Ministry of Health, PO box 7272, Kampala, Uganda; 3 Division of Malaria Control, Ministry of Health, PO Box 20750, Nairobi, Kenya; 4 Deceased

## Abstract

**Background:**

The accuracy of malaria diagnosis has received renewed interest in recent years due to changes in treatment policies in favour of relatively high-cost artemisinin-based combination therapies. The use of rapid diagnostic tests (RDTs) based on histidine-rich protein 2 (HRP2) synthesized by *Plasmodium falciparum *has been widely advocated to save costs and to minimize inappropriate treatment of non-malarial febrile illnesses. HRP2-based RDTs are highly sensitive and stable; however, their specificity is a cause for concern, particularly in areas of intense malaria transmission due to persistence of HRP2 antigens from previous infections.

**Methods:**

In this study, 78,454 clinically diagnosed malaria patients were tested using HRP2-based RDTs over a period of approximately four years in four highland sites in Kenya and Uganda representing hypoendemic to mesoendemic settings. In addition, the utility of the tests was evaluated in comparison with expert microscopy for disease management in 2,241 subjects in two sites with different endemicity levels over four months.

**Results:**

RDT positivity rates varied by season and year, indicating temporal changes in accuracy of clinical diagnosis. Compared to expert microscopy, the sensitivity, specificity, positive predictive value and negative predictive value of the RDTs in a hypoendemic site were 90.0%, 99.9%, 90.0% and 99.9%, respectively. Corresponding measures at a mesoendemic site were 91.0%, 65.0%, 71.6% and 88.1%. Although sensitivities at the two sites were broadly comparable, levels of specificity varied considerably between the sites as well as according to month of test, age of patient, and presence or absence of fever during consultation. Specificity was relatively high in older age groups and increased towards the end of the transmission season, indicating the role played by anti-HRP2 antibodies. Patients with high parasite densities were more likely to test positive with RDTs than those with low density infections.

**Conclusion:**

RDTs may be effective when used in low endemicity situations, but high false positive error rates may occur in areas with moderately high transmission. Reports on specificity of RDTs and cost-effectiveness analyses on their use should be interpreted with caution as there may be wide variations in these measurements depending upon endemicity, season and the age group of patients studied.

## Background

Most countries in sub-Saharan Africa now recommend first-line treatment of malaria with artemisinin-based combination therapy (ACT). These combinations are highly effective against drug-resistant *Plasmodium falciparum*, but are substantially more expensive than previously used drugs. Currently, ACT antimalarials are made available in many countries through external support, such as that provided by grants from the Global Fund to Fight AIDS, Tuberculosis and Malaria. However, their high cost means that their rational use is essential to ensure sustainability.

Malaria treatment in most endemic countries in Africa is based on clinical signs and symptoms due to lack of reliable microscopy in the majority of peripheral health units. The use of rapid diagnostic tests (RDTs) in malaria diagnosis is, therefore, increasing in many countries as the result of their ease of use with minimal training [1]. In the face of increasingly expensive malaria treatment regimens, the introduction of RDTs in peripheral health units is being advocated as a means of avoiding over-diagnosis of malaria. Compared with presumptive treatment, RDTs have been reported to be cost-effective in most parts of Africa and they may be beneficial in reducing inappropriate treatment of non-malarial febrile illnesses, in particular bacterial infections [2].

The diagnostic accuracy of RDTs can vary substantially across different geographical areas making it difficult to compare results from studies conducted under non-standard conditions [3]. RDTs that detect the histidine-rich protein 2 (HRP2) antigen (which is uniquely synthesized by *P. falciparum*) have been recommended in endemic areas where this species is dominant, due to their relatively low cost, high sensitivity and stability [4]. An alternative type of RDT detects the enzyme parasite lactate dehydrogenase (pLDH) which is produced by all four human *Plasmodium *species. Although HRP2-based tests are generally more sensitive than pLDH-based tests, the relatively low level of specificity in diagnosing clinical malaria of HRP2-based tests is a cause for concern [5]. This reflects the fact that HRP2 can persist in the blood stream for several weeks, resulting in high false positive error rates among patients with cleared parasitaemia who seek treatment for illnesses other than malaria [6]. A high number of false positives can compromize the cost-effectiveness of these tests.

There is little information on the epidemiological factors that influence the specificity of HRP2-based tests. In the present study, we assessed the sensitivity, specificity, positive predictive value (PPV) and negative predictive value (NPV) of RDTs compared with expert microscopy in two different malaria transmission settings. Diagnostic performance was analysed by age group, month of presentation and according to a number of patient-related characteristics and a statistical model was used to investigate the effects of different factors on false positive error rates. Differences in the positivity rates of RDTs across highland sites with varying transmission levels and in different months were also investigated. The implications of the findings are explored in relation to disease management in different areas with varying endemicity. Possible ramifications of the findings in terms of interpretation of cost-effectiveness analyses and accuracy of malaria surveillance data generated from the use of HRP2-based tests are discussed.

## Materials and methods

The results reported in this paper were obtained during the course of two studies undertaken as components of a large epidemic surveillance project conducted in four highland districts of Kenya and Uganda [7,8].

### Longitudinal study

One of the two studies involved longitudinal monitoring of factors influencing malaria transmission undertaken in four sites at varying altitude where meteorological, entomological, clinical and parasitological data were collected concurrently over a period of approximately four years. The two sentinel sites in Uganda were Bufundi Health Centre (29°52' E, 1°17'S; elevation 2291 m) in Kabale District and Kebisoni Health Centre (30°01' E, 0°51' S; elevation 1670 m) in Rukungiri District. Bufundi has a cool climate whereas Kebisoni is characterized by a mild climate. Both areas have two rainy seasons: March-April and September-November. Temperatures are highest in February and between June and August. Many inhabitants in Bufundi are subsistence farmers who often also travel to neighbouring districts to work as migrant labourers in large farms. Inhabitants of Kebisoni are relatively sedentary farmers. The two study sites in Kenya were Sengera and Kilibwoni Health Centres, located in Gucha and North Nandi Districts, respectively. Sengera (34°43'E, 0°52'S; elevation 1816 m) is the only non-governmental health centre among the four sites and has a mild climate. Kilibwoni (35°14'E, 0°13'N; elevation 2065 m) is characterized by a cool and wet climate suitable for growing tea, which is a significant cash crop in the area. In both Kenyan study areas, peak temperatures occur during February and March and the two rainy seasons are April-June and September-December.

At each study health centre, patients diagnosed clinically as a case of malaria according to standard national procedures for case management were subsequently tested using the Paracheck Pf^® ^test (Orchid Biomedical Systems, Goa, India) over approximately four years between November 2002 and September 2006 (except in Gucha where the study terminated in May 2006). The devices were stored at room temperatures within the range recommended by the manufacturer and used within the duration of the recommended shelf life of 24 months. The proper storage and use of the devices were ensured by supervisory staff at each site. The devices were purchased six times during the study period directly from the same manufacturer at the same time for both countries, but no attempt was made to use similar batches in all sites at similar times due to substantial variations in rates of use between sites and due to unpredictable nature of patient numbers. In a few cases, devices stored at sites with lower rates of use were transferred to other sites with higher rates. Clinical diagnosis of malaria was made usually by clinical officers on the basis of presence of fever or history of fever and absence of any other obvious cause of fever. Ethical approval for the study was obtained from relevant authorities in each country and from the London School of Hygiene and Tropical Medicine. Laboratory staff were given training on how to undertake RDT tests and on how to interpret the test results according to the manufacturer's instructions. Patients were asked by laboratory staff about any history of travel during the previous two weeks and about the use of antimalarials prior to the visit. Axillary temperature was measured using a digital thermometer. All patients diagnosed clinically as malaria cases were treated according to national guidelines irrespective of the outcome of the RDTs. The antimalarials used at the time of the study were a chloroquine and sulphadoxine-pyrimethamine (SP) combination in Uganda, and SP and later amodiaquine in Kenya.

### Studies of the sensitivity and specificity of RDTs

Studies to determine the sensitivity and specificity of the HRP2-based RDTs in comparison with expert microscopy were carried out at Kebisoni (Uganda) and Kilibwoni (Kenya) between December 2005 and March 2006 (a transmission season in both sites). These sites were selected because of their similarly sedentary populations and the fact that they were both government facilities, while they differed markedly in altitude and malaria endemicity. Finger-prick blood samples were taken from all clinically diagnosed malaria cases for both microscopic examination and RDTs. Verbal consent of patients or guardians was requested before taking blood samples. Thin and thick blood films were prepared and filter paper samples obtained for molecular studies (not reported in the present paper). Giemsa staining was used according to standard procedures. Slides were examined until 200 white blood cells (WBCs) were counted if positive. Slides for which parasites were not detected after counting up to 200 WBCs were examined until 400 WBCs were counted before a slide was considered to be negative. Parasite density per microlitre of blood was estimated by multiplying the counts by 8,000 (the approximate number of WBCs per microlitre) and dividing the result by the WBC counts. Duplicate slides were examined independently by two experienced microscopists who were blinded to the RDT results. In the case of a discrepant result, a third microscopist re-examined both slides. Results from the third microscopist were considered final.

### Data analysis

Microsoft Access version 2000 (Microsoft Corporation, Seattle, USA) was used for data entry. Stata Version 10 (StataCorp, College Station, Texas, USA) was used for data analysis. The sensitivity, specificity, PPV and NPV of the RDTs were compared between the two areas, across different age groups and months covering the transmission period. A logistic regression model was used to study the effects of variations in patient characteristics (sex, age, presence of fever, travel history, prior intake of antimalarials as reported by patient or guardian, and prior visit to the health facility), month of presentation, and geographical area on the specificity of RDTs. The variable of interest was false positive RDT test results among microscopically confirmed negative tests.

## Results

### Variations in RDT positivity rate by site

**Figure 1:**
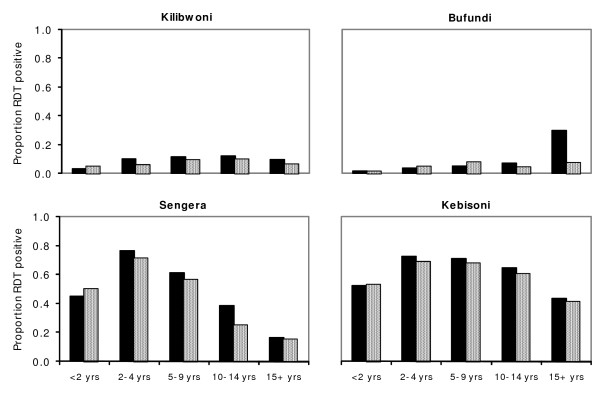
RDT positivity rates at four sentinel sites in Uganda and Kenya, October 2002 – September 2006, by gender and age group (key: dark and grey bars represent males and females, respectively).

A total of 78,454 patients with a clinical diagnosis of malaria were tested using RDTs over a period of approximately four years at four study health centres; 25,473 (32.5%) tested positive for *P. falciparum *malaria. Bufundi and Kilibwoni, both located at relatively high altitude, had lower RDT positivity rates compared to Kebisoni and Sengera (Table 1). Positivity rate increased with decreasing altitude. Sites located at high altitudes showed similar positivity rates among all age groups (Figure 1) except for increased rates in males aged 15 years and above at Bufundi, which probably reflects high levels of mobility in this group due to seasonal labour in neighbouring (and more endemic) districts. Some variations in morbidity levels between age groups were observed in Sengera and Kebisoni, areas located at lower altitudes. Sengera, which is a non-governmental health centre, showed an age pattern compatible with moderately high endemicity in which relatively few adults are affected compared with younger age groups.

**Table 1:** Altitude, annual climate, malaria incidence rates (estimated from the number of RDT-positive cases who were residents of the locality where each health centre is located and using the population of the locality as denominator) and RDT positivity rates at the four sentinel sites in Uganda and Kenya*.

Site	Altitude (m)	Average temperature (°C)	Average annual rainfall (mm)	Malaria incidence rates per 1000 per year	Overall RDT positivity rate (%)
Bufundi, Uganda	2291	16.1	884	15.6	5.8
Kilibwoni, Kenya	2065	17.0	1,424	43.2	7.9
Sengera, Kenya	1816	18.9	1,709	3.4	42.2
Kebisoni, Uganda	1670	20.1	1,007	359.8	52.3

*Patients who had a travel history in the two weeks before the tests were done were excluded from the data presented in the table.

**Figure 2:**
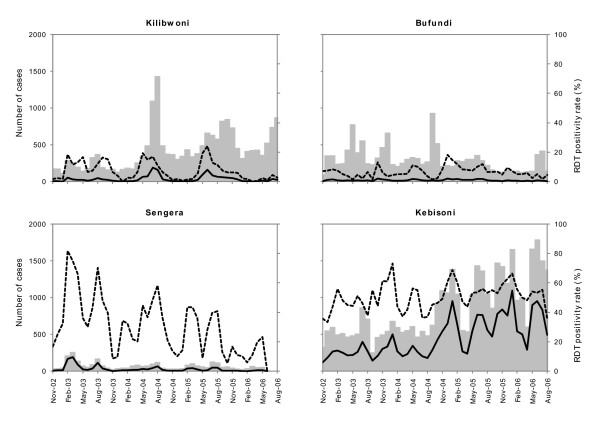
**Longitudinal variations in number tested (grey bars), RDT positive cases (solid line) and the corresponding RDT positivity rates (dashed line) at four sites in Kenya and Uganda between November 2002 and August 2006.** Patients with a travel history in the previous two weeks before presentation were excluded. All patients clinically diagnosed as malaria cases were subsequently tested with RDTs, except in Kilibwoni between January 2003 and February 2004 when approximately 50% were tested.

RDT positivity rates varied by season and year at each site, indicating temporal changes in accuracy of clinical diagnosis of malaria (Figure 2). The absolute number of suspected cases of malaria who tested positive varied between sites depending on altitude and type of health facility. As an example, Sengera, the non-governmental facility, charged fees for consultation and drugs whereas the other government facilities provided free treatment, resulting in relatively low observed attendance at the facility.

RDT positivity rates increased as the number of RDT-positive cases increased, especially in sites located at lower altitudes. There was a strong correlation between monthly RDT positivity rates and number testing positive with RDTs in all sites, with correlation coefficients varying between 0.64 (Kebisoni) and 0.87 (Sengera). At Kebisoni, both clinical malaria cases and RDT-positive cases increased during the study period, but there was no similar trend in the RDT positivity rate (Figure 2).

### Accuracy of RDTs compared to expert microscopy

**Figure 3:**
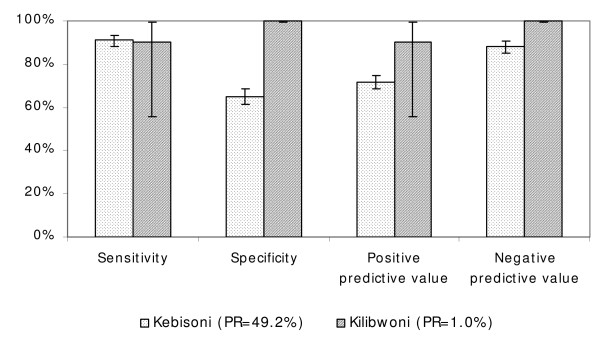
**Sensitivity, specificity, PPV and NPV of RDTs compared to microscopy in Kebisoni (mesoendemic area) and Kilibwoni (hypoendemic area).** Error bars indicate 95% confidence intervals.

At the hypoendemic site (Kilibwoni), only 10/1,000 (1.0%) of cases examined microscopically were positive *for P. falciparum *by RDT, whereas at the mesoendemic site (Kebisoni), 609/1,237 (49.2%) were positive. The sensitivity, specificity, PPV and NPV of the RDTs at Kilibwoni were 90.0%, 99.9%, 90.0% and 99.9%, respectively, whereas the corresponding figures at Kebisoni were 91.0%, 65.0%, 71.6% and 88.1%, respectively. A significantly higher specificity was observed at Kilibwoni compared to that of the more endemic Kebisoni (*p *< 0.0001) (Figure 3). This resulted in a significantly higher NPV for RDTs in the former (*p *< 0.0001), but there was no significant difference between the two sites in terms of PPV (*p *= 0.198). At Kebisoni, 220/628 patients (35%) who tested negative by microscopy tested positive by RDT. At Kilibwoni, only one of the 990 patients who tested negative by microscopy tested positive by RDT. Fifty-five of the 609 patients (9%) confirmed to be positive with microscopy at Kebisoni were declared negative with RDTs. Most of these patients had low mean parasite densities (below 1,000/μl in 34/55). However, six of the 55 false negative patients at Kebisoni (11%) had parasite densities exceeding 8,000/μl. At Kilibwoni, one patient was false negative by RDT out of a total of 10 who were confirmed positive by microscopy.

**Figure 4:**
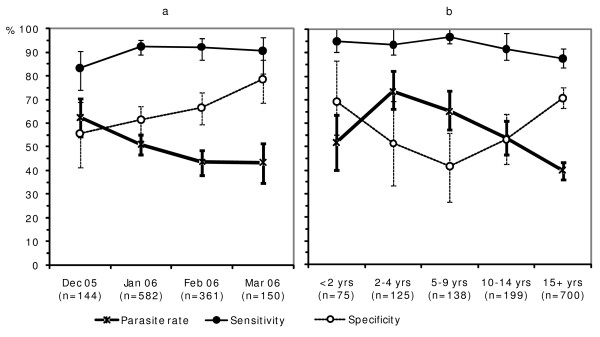
Sensitivity and specificity of RDTs as a function of the true parasite rate (as determined by microscopy) at Kebisoni, Rukungiri District, Uganda, by (a) month and (b) age groups (error bars indicate 95% confidence intervals).

At Kebisoni, true parasite rates (as determined by microscopy) declined during the four months (December 2005 – March 2006) of concurrent collection of blood samples for comparison of RDTs with microscopy. During this period, the specificity of RDTs increased steadily from 56% in December 2005 to 79% in March 2006 (Figure 4a). There was no substantial change in the sensitivity of RDTs. During the same period, the NPV of RDTs increased from 67% to 92% whereas there was little change in PPV (76% in December 2005 and 77% in March 2006).

The true parasite rate varied between age groups. The peak parasite rate was observed in children 2–4 years of age and the rate decreased in the older age groups (Figure 4b). Specificity of RDTs increased as parasite rates decreased, but sensitivity was more or less uniform among the various age groups.

**Figure 5:**
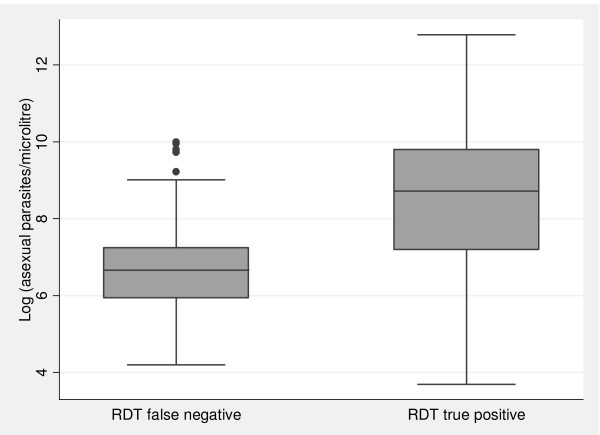
Differences in parasite densities between false negative and true positive RDT results compared to microscopy at Kebisoni, Uganda.

Sensitivity of RDTs was significantly higher in patients with fever (body temperature of 37.5°C and above) on presentation compared to non-febrile patients (97% versus 89%, *p *= 0.006) but specificity was significantly lower in febrile patients (33% versus 69%, *p *< 0.0001). No significant differences were detected between the two groups in terms of PPV and NPV (*p *= 0.827 and *p *= 0.742, respectively). At Kebisoni, microscopically confirmed *P. falciparum *patients with high parasite densities were significantly more likely to be true positive with RDTs than patients with a low parasite density (Figure 5). The mean parasite densities of false negatives and true positives were 898/μl and 5,215/μl and this difference was statistically highly significant (*p *< 0.0001).

A logistic regression model showed that age, presence of fever, area and month of presentation were significantly and independently associated with probability of a negative RDT test result being a true negative (Table 2). False positive error rates declined in older age groups. Patients with fever at the time of presentation were more likely to test false positive with RDTs compared to those without. The site at higher altitude and with low malaria transmission intensity was associated with higher specificity. Specificity increased towards the end of the transmission season. Previous intake of antimalarials, revisit in the previous two weeks, travel outside the district in the previous two weeks and sex were not significantly associated with the probability of a negative RDT test result being true negative.

**Table 2:** Outputs of the best-fitting logistic regression model for factors associated with the probability of obtaining true negative HRP2-based RDT test results at Kebisoni, Uganda.

Factors	Odds ratio	Standard error	*P*
Area (Kebisoni relative to baseline = Kilibwoni)	0.002	0.002	< 0.0001
Age (years)	1.017	0.005	0.002
Presence of fever at the time of presentation (relative to baseline = absence of fever at the time of presentation)	0.275	0.073	< 0.0001
January (relative to baseline = December)*	1.173	0.367	0.609
February (relative to baseline = December)*	1.414	0.458	0.285
March (relative to baseline = December)*	2.623	1.026	0.014

Previous intake of antimalarials, a clinic visit in the previous two weeks, travel outside the district in the previous two weeks and sex were not significantly associated with the dependent variable.

* Significance of the combined effect of months: Chi-squared at 3 degrees of freedom = 8.57, *p *= 0.0356.

## Discussion

This study showed that in an area with moderate malaria transmission, more than a third of patients with positive HRP2-based RDT tests had a negative blood film and may have been incorrectly diagnosed as a case of clinical malaria due to persistence of the HRP2 antigen from an earlier infection. It is possible that a few patients with submicroscopic or low levels of parasitaemia might have been wrongly classified as negative with microscopy. However this number is likely to have been very small as blood films were read carefully by two experienced microscopists. In contrast, nearly all RDT positive patients in a site with low endemicity were true positives. False positive error rates declined with increasing age of patients, probably as the result of acquired immunity in clearing parasite antigens. Previous studies have shown that HRP2-based RDTs can lead to high false positive error rates. Swarthout *et al *reported that by using Paracheck-Pf^®^, 73% of cases were still RDT test positive 35 days after treatment and that the false positive error rate correlated with initial parasite density [6]. Iqbal *et al *found nearly 35% of patients still had HRP2 antigenaemia 14 days after treatment despite negative blood films [9]. In another study, 61% of patients had positive HRP2-based RDT tests for more than two weeks after initiation of treatment [10]. These antigens are eventually cleared by anti-HRP2 antibodies, especially anti-HRP2 IgG [11].

The study also showed that the specificity of RDTs varied seasonally in the same area. At the mesonendemic site (Kebisoni), specificity increased as the true parasite rate (as determined by microscopy) decreased at the end of the transmission season. This may have followed from boosting of anti-HRP2 antibodies as a result of infections acquired during the preceding few months. The relatively higher specificity of RDTs at the hypoendemic site compared with the mesoendemic site could, on the other hand, be due to a very low probability of finding patients with recently cleared parasitaemia who sought treatment for non-malarial illnesses.

Sensitivity of RDTs was not affected by age of patient or fluctuation in parasite rates during different months. It was, however, affected by parasite density. Patients with high parasite densities were more likely to test positive than those with low parasitaemia. Other studies have also indicated that HRP2-based tests have high sensitivity which increases with parasite density [3]. However, sensitivity can vary from area to area. There are variations in the repeat section of the HRP2 protein between parasite isolates from different areas which might be a reason for wide variations in sensitivity of HRP2-based RDTs in different areas [12]. A study carried out in Uganda showed that the PPV was only 20% at a site of low endemicity (in Kabale District) whereas in other areas with higher endemicity it was much higher, while the NPV was uniformly high (> 97%) [13]. In the present study, estimates of both the PPV and NPV were high at the site with low endemicity in Kenya. Although it has been suggested in one study that sensitivity is affected by age-dependent immune status of patients independent of parasite density [14], no evidence of this phenomenon was found in the present study.

It might be argued that some of the variability observed between the sites and during different parts of the year could have resulted from performance variability of the tests used, especially as climatic conditions affect the stability of the devices. Stability is usually more problematic with pLDH-based tests than with HRP2-based tests. Due to variable rates of use in different sites, the use of similar batches of the tests across all sites and seasons could not be ensured. Nevertheless, the devices were purchased at the same times for both countries, and from the same manufacturer during the entire study period. The devices were also stored and used within the recommended temperature and duration. Due to these and the fact that the sensitivity of the tests in Kenya and Uganda were similar (90.0% and 91.0%, respectively), variability in performance of the devices is unlikely to have played a major role.

The use of RDTs is probably cost-effective in many situations. A simulation study has indicated that at a 95% confidence level, RDTs are cost-effective compared to presumptive treatment below 62% parasite prevalence rates [2]. However, cost-effectiveness of RDTs can be compromized if patients with negative RDT tests are prescribed antimalarials [15] as has been shown in both Tanzania [16] and Zambia [17] to frequently be the case. A danger of reliance on RDTs is that some patients who require malaria treatment may test negative and be given symptomatic treatment only. In this study, the fact that there were 9% false negative RDT tests among microscopically confirmed cases at Kebisoni, some of whom had high parasitaemia, shows the risk of relying on test results alone. Due to potential variations in the accuracy of RDTs by season, as suggested by the present study, seasonal use of these diagnostic tools may be necessary after careful cost-effectiveness studies in some areas, especially those with mesoendemic transmission.

During an epidemic caused by flooding in Mozambique, RDTs were shown to have an adequate PPV when combined with clinical diagnosis, although they failed to detect some true malaria cases [18]. Thus, the use of RDTs for treatment decisions in epidemics could increase the risk of missed treatment. Furthermore, the cost-effectiveness of using RDTs during epidemics is unclear. For example, one study showed that the percentage of confirmed malaria cases must not exceed 55% for RDTs to be cost-effective when artemether-lumefantrine is used for treatment [19]. The threshold level for artesunate-amodiaquine was even lower (21%). During epidemics, the proportion of fever cases who test positive can increase considerably in a short period of time.

Although the use of RDTs for treatment decisions in epidemic situations may be limited, their use can be helpful in surveillance, for example in the confirmation of reported outbreaks. However, RDT-based longitudinal data should be interpreted with caution due to potential seasonal or annual variations in accuracy resulting from temporal changes in transmission levels as indicated in this study. The study also shows that data generated from clinical diagnosis alone could be useful in epidemic monitoring, in particular in mesoendemic situations. However, in highlands with very low endemicity, the use of clinical data alone without laboratory confirmation can be misleading. This was observed in Bufundi, in Uganda, where an apparent outbreak of clinical malaria was confirmed to be a non-malarial febrile illness [8].

One of the major implications of the findings of the present study is that cost-effectiveness of HRP2-based RDTs is greatly influenced by variations in their sensitivity and specificity between different areas, age groups of patients, and seasons. The study showed that the diagnostic accuracy of HRP2-based RDTs is relatively high in areas or seasons with low transmission, but more area-specific operational studies may be required to evaluate their cost-effective use under different transmission scenarios. For decisions involving the use of these tests, policymakers should take into account the cost implications of treating test negative patients [15], as well as the risk of not treating false negative patients. The cost-effectiveness of HRP2-based RDTs depends on a multitude of factors: overall diagnostic accuracy, prevalence and its seasonal fluctuations, seasonal changes in test specificity, age group of study subjects, parasite density, the relative cost of antimalarials and RDT tests, the relative treatment costs of test negative cases and the extent to which clinicians trust the outcomes. RDT test results should always be interpreted together with clinical assessment of the patient, allowing for fallibility of the devices [20]. In some vulnerable patients (e.g. children), the risk of leaving a false-negative case untreated for malaria may outweigh the costs of over treatment based on clinical diagnosis [4]. Authorities in charge of developing malaria diagnostic policies may have to interpret reports on specificity of HRP2-based RDTs and cost-effectiveness analyses on their use with some caution as there may be wide variations in the determinant factors of accuracy between different studies. In some areas, it may be useful to vary the use of HRP2-based RDTs according to factors such as transmission level, season and age group of patients, but such policies should be based on further area-specific investigations. Especially in situations where the diagnostic accuracy of RDTs is unlikely to be high, health services will need to strengthen microscopy.

## Authors' contributions

TAA and JC conceived and designed the study. TAA coordinated the study, trained field staff, compiled data, carried out statistical analyses and wrote the manuscript. MK trained field staff, contributed to planning of the sample collection, assisted in data compilation and participated in revision of the draft. JC and BG revised the draft and facilitated funding of the project. CJ contributed to discussions on manuscript ideas and preliminary exploration of the data, and commented on the draft. JB developed and maintained the database used in the study. JB and DM contributed to revision of the manuscript. MO and BR coordinated the field studies in Uganda and Kenya, respectively. All authors (except BR who sadly passed away) read and approved the final manuscript.
